# Dietary self-efficacy and social support interactions in junior athletes' acquisition of life skills

**DOI:** 10.3389/fspor.2022.673633

**Published:** 2022-09-28

**Authors:** Yuka Shudo, Kazuho Yamaura, Jun Yasuda, Ai Sato, Kumiko Ebi

**Affiliations:** ^1^Graduate School of Sport and Health Science, Ritsumeikan University, Kusatsu, Japan; ^2^College of Sport and Health Science, Ritsumeikan University, Kusatsu, Japan; ^3^Japan Institute of Sports Sciences, Tokyo, Japan

**Keywords:** social skills (MeSH), self-efficacy, social support, adolescent, nutrition education

## Abstract

**Objective:**

According to the stages of change, this study identified the association between dietary control self-efficacy and social support for healthy diets as factors influencing life skills acquisition in dietary habits among adolescents in Japan.

**Research design:**

This cross-sectional study was conducted between April and May 2018 among eight high school baseball teams in Japan.

**Method:**

Participants included 180 Japanese high school baseball players. Survey items evaluated life skills (dependent variables), self-efficacy's influence on dietary control, social support (explanatory variables), and stages of change. Hierarchical multiple regression analysis was used to reveal the associations.

**Results:**

In the pre-action stage, although there was no significant association between the interaction of self-efficacy and social support on total life skills (β = 0.11, *p* = 0.158), a significant association was observed in the action/maintenance stage (β = 0.32, *p* < 0.05). The interaction between self-efficacy and social support showed a significant association with goal setting in the pre-action stage (β = 0.19, *p* < 0.05) and with communicating in the action/maintenance stage (β = 0.34, *p* < 0.05).

**Conclusion:**

The acquisition of life skills amidst dietary situations can be facilitated by providing social support that considers self-efficacy in relation to dietary control, according to the stages of change.

## Introduction

Acquiring life skills is essential for promoting healthy development among children and adolescents (1). The World Health Organization (2) defines life skills as “abilities for adaptive and positive behavior that enable individuals to deal effectively with the demands and challenges of everyday life.” In dietary habits, complex information (e.g., differences in nutritional value and safety of foods) should be analyzed and evaluated when selecting what an individual needs from various foods (3). In this process, various life skills such as assertiveness, self-management, negotiation, and critical thinking are needed (3), and therefore, the dietary habits should contain enough elements for acquiring those skills. To our knowledge, however, empirical research on the relationship between diet and life skills is insufficient. Therefore, this study focused on the educational strategy of food and nutrition-related aspects in the context of sports to promote acquisition of life skills among adolescent athletes. The relationships among the factors involved in the acquisition of life skills through food and nutrition should be elucidated to develop life skills education related to dietary habits.

The theoretical framework of Social Cognitive Theory (SCT) helps consider these relationships. The SCT states that personal, environmental, and behavioral factors interact with each other (4). Most of the behavioral factors that comprise SCT are controlled through self-regulatory functions (4), including life skills concepts such as goal setting. In this study, we used self-efficacy on dietary control (hereafter “DSE”) as a personal factor and social support on healthy diets (hereafter “DSS”) as an environmental factor to clarify the relationship between these factors (DSE and DSS) and life skills as a behavioral factor. In terms of the relationship between life skills and self-efficacy (hereafter “SE”), Bandura and Wood (5) states that SE has a strong influence on personal goal setting. Furthermore, Sheard and Golby (6) reported that training in psychological skills, including goal setting, contributes to increased SE. In a study, the group whose DSE improved through nutrition education also showed greater improvement in life skills (7). Thus, life skills and DSE are expected to be interrelated. A study (8) demonstrating the relationship between life skills and social support (hereafter “SS”) found that for athletes, building relationship with their coaches and development of their life skills were related. More specifically, athletes, perceiving that higher levels of coaching behaviors were related to life skills including goal setting, had a positive rapport with their coaches and viewed their experiences of sports positively. On the other hand, those who reported negative rapport with their coaches, experienced stress through their sports participation. These results suggest that building positive SS rapports with coaches is important for athletes' life skills development.

While targeting adolescents who are in the developmental stages both mentally and physically, an individual's stage of behavioral change should be considered. Therefore, the relationship between the factors also needs to be clarified according to the stage of behavioral change. In this study, the Transtheoretical Model (TTM) (9) based on time dimension, was used to understand the stages of change. The five stages of change are defined as: precontemplation i.e., “not intending to take action in the foreseeable future, usually measured as the next 6 months;” contemplation i.e., “intending to change in the next 6 months;” preparation i.e., “intending to take action in the immediate future, usually measured as the next month;” action i.e., having “made specific overt modifications in their life styles within the past 6 months;” and maintenance i.e., “working to prevent relapse,” with this stage continuing between ~6 months and 5 years (9). The model states that because the stages of readiness to change health behaviors differs across individuals, the components and processes that move them to act are qualitatively different (10). Specifically, in the early stages, individuals rely on cognitive, emotional, and appreciative processes (10). In contrast, in action-oriented stages, individuals increasingly draw upon commitments, conditioning, coincidences, environmental management, and support to move toward maintenance or termination of behavior (10). In other words, while personal factors are the main elements of the process in the early stages, environmental factors are considered main in the action-oriented stages. In this study, the early stage is described as the “pre-action stage” (precontemplation, contemplation and preparation) and the action-oriented stage as the “action/maintenance stage” (action and maintenance). A comparative study using the stages of change of TTM in dietary habits showed that those in the action stage have higher self-efficacy of eating behaviors than those in the pre-action stage (11). Benight et al. (12) states that high SE helps in effectively managing SS, suggesting that, during the action/maintenance stage, the effective use of SS may depend on SE. In dietary habits, studies examining psychosocial factors or dietary behaviors based on the stages of change have focused on foods and dietary behaviors such as promoting increased vegetable and fruit intake (13–15) or decreasing obesity (16–18), indicating the importance of DSE and support from those around the person. Regarding dietary habits, a study using the SCT as a theoretical framework in adults (19) found that social support indirectly predicted dietary behavior through self-regulation and self-efficacy, while self-efficacy indirectly affected dietary behavior through self-regulation. However, insufficient studies have been conducted to identify the relationships among these variables by stages of change to develop life skills in adolescents. Since life skill acquisition is particularly important during childhood and adolescence (1), there is a strong need to clarify the relationship among factors affecting life skills at different stages of change. Therefore, this study aimed to examine the association between DSE and DSS as factors influencing adolescents' life skills acquisition in dietary situations according to the stages of change.

We formulated two hypotheses:

***Hypothesis 1***: DSE and DSS are associated with and affect life skills acquisition.

***Hypothesis 2***: Depending on the stages of change, DSE will be the key factor associated with the pre-action stage, whereas DSS will be the main influence during the action/maintenance stage, for life skills acquisition.

## Materials and methods

### Research design

This cross-sectional study was conducted during April–May 2018 among high school baseball players in Japan.

### Setting and participants

Participants were 180 first-year high school baseball players from eight high school baseball teams. Seven of these eight teams belonged to public schools, and one team was from a private school. All participants were day scholars. To recruit the participants, we approached the target teams' teachers, who then informed the respective players. We then, in writing, clarified our research objectives, investigation details, and the potential disadvantages to the prospective participants and their legal guardians, after which only those who were willing to participate and provide their consent were included in the study. More specifically, they were apprised that participation was optional and could be withdrawn, even after providing consent, through a written withdrawal of consent. Those who agreed to participate provided written informed consent. Notably, no school provided nutrition education to participants between admission and the time of the survey. The survey was self-administered, and the study ultimately involved 179 participants (15.1 ± 0.3 years old), excluding one who did not respond to the stages of change component. The study protocol was approved by the Ritsumeikan University Ethics Review Committee for Medical and Health Research Involving Human Subjects and was conducted per the Declaration of Helsinki.

#### Questionnaire

The survey items were related to life skills as the dependent variables, and DSE and DSS as explanatory variables. The stages of change helped identify the dietary behavior change process. The scales used to assess life skills and DSE were developed for college student-athletes, and the life skills scale has also been used with high school student-athletes (20). Thus, these scales were used for the high school student-athletes in this study.

First, we evaluated life skills using a scale for athletes (21) previously found reliable and valid. Responses to each question were scored from 1 (not applicable at all) to 8 (very applicable). A confirmatory factor analysis (CFA) was performed with 40 items (e.g., I communicate with every member of my team) and 10 subscales. Convergent validity was assessed using the means of composite reliability (CR), factor loading (FL), and average variance extracted (AVE). All the CRs for each construct were greater than the recommended value of 0.60 (22), except that for “Maintaining physical health and wellbeing,” which was 0.57. The FLs of all the subscales were >0.5 (23), except that for “Maintaining physical health and wellbeing,” which was 0.499. Moreover, all the AVEs were greater than the recommended value of 0.5 (24), except those for “Thinking carefully,” “Taking responsibility for one's own behavior,” and “Maintaining physical health and wellbeing,” which were 0.44, 0.49, and 0.26, respectively. These three subscales were judged to have low convergent validity but were validated otherwise. Internal consistencies of the life skills scales were examined by computing the Cronbach's alpha coefficient for each subscale; the results reported that except for “Maintaining physical health and wellbeing” (alpha = 0.58), all the other nine subscales had alpha values of 0.70 or higher. The goodness of fit: χ^2^ =1087.08, df = 695, *p* < 0.001, GFI = 0.78, AGFI = 0.74, RMSEA = 0.06, AIC = 1,337.08. As some scale items did not meet all the validity and reliability criteria, it may have been necessary to either delete those items or synthesize them with items on other similar scales, as is commonly done, in order to improve them. However, as pointed out by Haebara (25), the deletion or synthesis of items may reduce the generalizability of the scale measurements for use in subsequent related studies. Therefore, although we recognize that some of the scales we tested may not meet our validity and reliability criteria, we opted to retain them in recognition of their generalizability for future studies in various populations.

Second, DSE was evaluated through a self-efficacy scale on dietary control (26), comprising 19 items (e.g., I can eat a well-rounded diet with staple food, main dishes, and side dishes even when I am alone) structured under five subscales. The scale has been found to be reliable and valid (26). Responses were scored from 1 (not confident) to 8 (confident). The total DSE score was used in this study to examine the relationship between comprehensive DSE and life skills. The CFA results for this subject confirmed that the CR was 0.94, which exceeds the minimum requirement of 0.60 for consistency (22). The FL was 0.67, which was >0.5 (23). The AVE, at 0.47, was close to 0.5 (24). The Cronbach's alpha coefficient for the DSE scale was 0.85, which reflects adequate internal consistency. The goodness of fit: χ^2^ = 275.13, *df* = 142, *p* < 0.001, GFI = 0.86, AGFI = 0.82, RMSEA = 0.07, AIC = 371.13. Although we find it problematic that the DSE scale did not fully meet the reliability and validity criteria, we chose, as with the life skills scale, to retain this instrument.

Third, DSS was evaluated through items based on the Functional Social Support for Healthy Diets scale (27). Its reliability and validity have been confirmed (27). The scale comprises a total of 12 items (three for each function, e.g., I have someone close to me who cooks healthy meals). The wording in one item was changed to fit the target population (from work environment to team environment). Each question was scored from 1 (*not applicable at all*) to 5 (*very applicable*). The CFA results for this subject confirmed that the CR was **0**.93, which exceeds the minimum requirement of 0.60 for consistency (22). The FL was 0.71 which was >0.5 (23). Moreover, the AVE was 0.54, which is deemed acceptable when >0.5 (24). The goodness of fit: χ^2^ =114.54, df = 48, *p* < 0.001, GFI = 0.91, AGFI = 0.85, RMSEA = 0.09, AIC = 174.54. To examine the relationship between comprehensive DSS and life skills, this study used the total score for DSS. The Cronbach's alpha coefficient for the DSS scale was 0.88, which reflects adequate internal consistency. All these assessments showed that this scale was appropriate for further analysis.

Fourth, the stages of change were evaluated through the TTM (9, 10). It includes five stages of change: precontemplation, contemplation, preparation, action, and maintenance. Since the present study used self-efficacy on dietary control, we used the question item on dietary-related stages of change (28). Respondents were asked to choose which of the five stages of change most applied to their current situation.

### Statistical analysis

Since some subscales of life skills were not normally distributed, Spearman's rank correlation coefficient was used to calculate the bivariate association between life skills, DSE, and DSS. Hierarchical regression analysis was performed using the forced input method. This procedure examined the degree to which DSE and DSS (and the interaction terms of both scales) predict the dependent variables, total life skills, and the subscale scores. Initially, DSE and DSS were added to Model 1. Subsequently, interactions between DSE and DSS were added to Model 2. A simple slope analysis was used to visualize the interaction term at one standard deviation above and below the mean and determine whether the standard partial regression coefficient in the interaction term was significant. Prior to the regression analysis, the variables in the model were standardized. We conducted the Breusch-Pagan test and the White test to assess homoscedasticity of the residuals. We conducted multiple analyses according to the combination of explanatory variables; thus, we conducted the two tests for each analysis. When the null hypothesis of homoscedasticity was rejected for either test with a 5% significance level, we adopted robust *t*-values to check the significance levels of the parameters. Otherwise, we adopted standard *t*-values. As a result, of the 11 analyses in each stage, only two showed heteroscedasticity in the pre-action stage (the cases of appreciating others and being humble) and one in the action/maintenance stages (the case of communicating). Consequently, heteroscedasticity was not considered to be a problem overall. The reason for the presence of heteroscedasticity is that our samples might be influenced by the characteristics of the eight targeted schools; however, the reason cannot be specified. Nonetheless, the estimated parameters are unbiased; thus, we believe that heteroscedasticity is not a serious problem for the purpose of this study as we explore only the signs of the estimated parameters. However, to assess the significance levels of the estimated parameters, we need to use robust *t*-values for the heteroscedasticity cases.

Notably, Prochaska et al. (10) outlined two types of stages—(a) the early stages, which exist before action, and (b) the action-oriented stages—that continuously emerge in the context of the relationship between stages and processes of change. Accordingly, this study divided the stage of change, depending on whether the action was performed continuously, into the following two stages: the pre-action stage (precontemplation, contemplation, preparation; *n* = 115) and the action/maintenance stage (action, maintenance; *n* = 64) and the relationships between the factors were examined. The threshold for significance was *p* < 0.05. Statistical analyses were performed using SPSS version 26.0 (IBM Corp., Tokyo, Japan, 2013).

## Results

### Descriptive statistics

The means, standard deviations, reliability coefficients, and correlations for DSE, DSS, life skills, and the 10 life skills subscales are described in Table 1. The Spearman's rank correlation coefficient revealed that DSE was positively associated with total life skills and all 10 life skills subscales (*r* range = 0.20–0.62, *p* < 0.01). The correlations revealed that DSS was positively associated with eight life skills and total life skills, excluding the skills of maintaining etiquette and manners and being humble (*r* range = 0.19–0.39, *p* < 0.05). Confirmed by the stages of change, the pre-action stage showed the same correlation as the overall result (*p* < 0.05). In the action/maintenance stage, DSE was significantly correlated with life skills, excluding maintaining etiquette and manners (*p* < 0.05), but DSS was not significantly correlated with total life skills or any subscales.

**Table 1:** Summary of intercorrelations, means, standard deviations, and reliability estimates.

		**Mean**	**SD**	**Cronbach's alpha**	**1**	**2**	**3**	**4**	**5**	**6**	**7**	**8**	**9**	**10**	**11**	**12**
1	Self-efficacy	99.5	18.2	0.86	–											
2	Social support	40.3	7.6	0.89	0.421***											
3	Life skills	243.2	27.6	0.91	0.624***	0.390***										
4	Stress management	22.3	5.8	0.89	0.384***	0.333***	0.560***									
5	Goal setting	17.8	6.6	0.84	0.326***	0.261***	0.517***	0.170*								
6	Thinking carefully	23.2	3.5	0.76	0.394***	0.187*	0.537***	0.372***	0.232**							
7	Appreciating others	27.0	4.4	0.85	0.359***	0.242**	0.679***	0.441***	0.243**	0.354***						
8	Communicating	24.2	5.0	0.83	0.285***	0.304***	0.581***	0.385***	0.122	0.279***	0.429***					
9	Maintaining etiquette and manners	29.3	4.9	0.93	0.203**	0.062	0.417***	0.106	0.135	–**0.0**17	0.204**	0.127				
10	Always making one's best effort	24.4	4.3	0.86	0.531***	0.243**	0.769***	0.267***	0.328***	0.441***	0.474***	0.357***	0.351***			
11	Taking responsibility for one's own behavior	27.4	3.5	0.79	0.408***	0.248***	0.727***	0.343***	0.239**	0.424***	0.521***	0.448***	0.274***	0.608***		
12	Being humble	24.6	4.3	0.81	0.353***	0.086	0.602***	0.117	0.171*	0.307***	0.381***	0.214**	0.405***	0.594***	0.501***	
13	Maintaining physical health and wellbeing	22.7	4.8	0.59	0.483***	0.288***	0.574***	0.217**	0.215**	0.157*	0.379***	0.219**	0.353***	0.381***	0.349***	0.266***

*p < 0.05.

**p < 0.01.

***p < 0.001.

### Factors related to life skills in the pre-action stage

**Figure 1:**
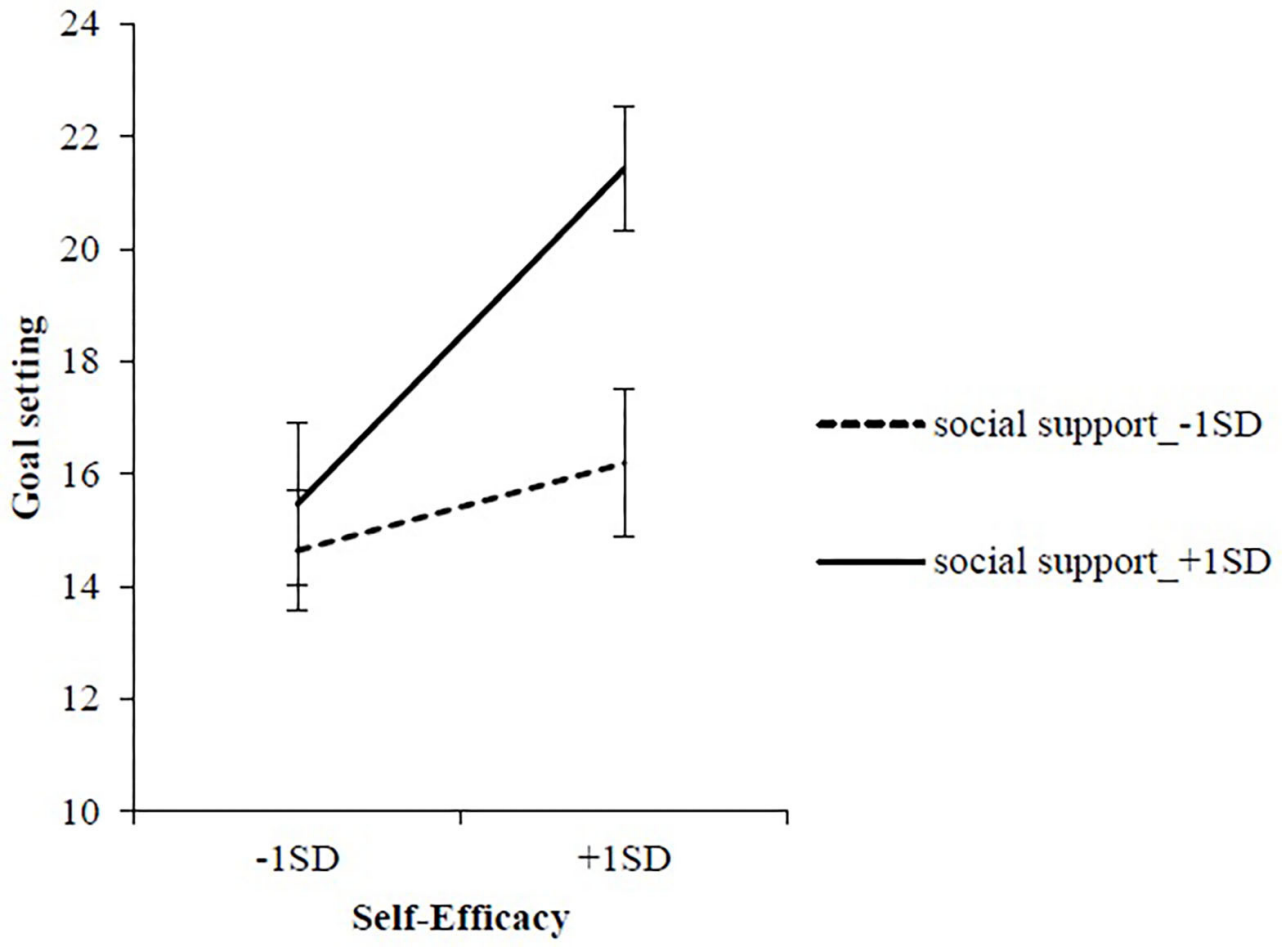
The interaction between self-efficacy and social support related to goal setting in the pre-action stage.

Table 2 presents the results of a hierarchical multiple regression analysis model examining the impact of DSE and DSS on life skills and its subscales during the pre-action stage. In Model 1, adding DSS and DSE resulted in a significant coefficient of determination (*R*^2^ = 0.42, *p* < 0.001); DSE and DSS were significantly positively associated with total life skills (DSE: β = 0.54, *p* < 0.001, DSS: β = 0.25, *p* < 0.01). In Model 2, adding the DSE and DSS interaction led to no increment in *R*^2^, and the interaction was not statistically significant. Upon performing the same steps with each subscale with life skills as the dependent variables, the increment in *R*^2^ in Model 2 was significant only for the goal-setting subscale (Δ*R*^2^ = 0.03, *p* < 0.05). The interaction term between DSE and DSS was also significant (β = 0.19, *p* < 0.05). To further examine the identified interaction effect, a simple slope analysis was used to depict the association between DSS and DSE in relation to goal setting. Results indicated that the goal-setting score was significantly higher at high DSE than at low DSE in situations of high DSS (1 standard deviation above the mean; *p* < 0.01). Conversely, in situations of low DSS (1 standard deviation below the mean), there was no significant difference between goal-setting scores at high and low DSE (*p* = 0.195; Figure 1). Additionally, the goal-setting score in high DSS situations was significantly greater than in low DSS situations only when DSE was high (*p* < 0.01; Figure 1).

**Table 2:** Hierarchical regression results of total life skills and subscales in the pre-action stage.

		**Total life skills score**					**Stress management**					**Goal setting**					**Thinking carefully**				
		**β**	***t*-value**	***R* ^2adj^**	** 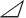 *R* ^2^**	***F*-value**	**β**	***t*-value**	***R* ^2adj^**	** 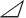 *R* ^2^**	***F*-value**	**β**	***t*-value**	***R* ^2adj^**	** 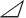 *R* ^2^**	***F*-value**	**β**	***t*-value**	***R* ^2adj^**	** 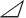 *R* ^2^**	***F*-value**
Step1	DSS	0.25**	3.37	0.42***	0.43***	40.60***	0.36***	4.20	0.24***	0.25***	18.37***	0.23*	2.56	0.13***	0.14***	9.16***	0.07	0.77	0.12***	0.14***	8.78***
	DSE	0.54***	7.28				0.27**	3.23				0.25**	2.71				0.35***	3.80			
Step2	DSS	0.27***	3.58	0.42***	0.01	27.99***	0.36***	4.22	0.23***	0.00	12.27***	0.27**	2.92	0.15***	0.03*	7.66***	0.07	0.80	0.11**	0.00	5.83**
	DSE	0.58***	7.42				0.29**	3.25				0.30**	3.20				0.35***	3.71			
	DSS × DSE	0.11	1.42				0.05	0.55				0.19*	2.04				0.03	0.26			
		**Appreciating others**					**Communicating**					**Maintaining etiquette and manners**					**Always making one's best effort**				
		β	***t*** **-value**	*R* ^2adj^	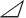 *R* ^2^	***F*** **-value**	β	***t*** **-value**	*R* ^2adj^	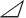 *R* ^2^	***F*** **-value**	β	***t*** **-value**	*R* ^2adj^	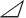 *R* ^2^	***F*** **-value**	β	***t*** **-value**	*R* ^2adj^	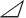 *R* ^2^	***F*** **-value**
Step1	DSS	0.10	1.09	0.22***	0.23***	16.91***	0.26**	2.87	0.10**	0.12**	7.20**	0.05	0.46	0.01	0.02	1.34	0.02	0.27	0.25***	0.26***	19.67***
	DSE	0.45***	4.47				0.16	1.72				0.14	1.42				0.51***	6.02			
Step2	DSS	0.11	1.33	0.22***	0.00	11.37***	0.28**	2.94	0.10**	0.00	4.94**	0.05	0.52	0.00	0.00	0.94	0.03	0.40	0.25***	0.00	13.25***
	DSE	0.47***	3.87				0.18	1.84				0.15	1.47				0.52***	5.99			
	DSS × DSE	0.06	0.47				0.07	0.71				0.04	0.40				0.07	0.75			
		**Taking responsibility for one's own behavior**					**Being humble**					**Maintaining physical health and wellbeing**									
		β	***t*** **-value**	*R* ^2adj^	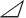 *R* ^2^	***F*** **-value**	β	***t*** **-value**	*R* ^2adj^	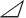 *R* ^2^	***F*** **-value**	β	***t*** **-value**	*R* ^2adj^	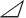 *R* ^2^	***F*** **-value**					
Step1	DSS	0.14	1.57	0.17***	0.19***	12.72***	−0.11	−0.94	0.11***	0.13***	8.21***	0.13	1.55	0.25***	0.26***	19.50***					
	DSE	0.38***	4.28				0.37***	3.44				0.46***	5.50							
Step2	DSS	0.14	1.56	0.16***	0.00	8.41***	−0.09	−0.80	0.11**	0.01	5.81**	0.13	1.51	0.24***	0.00	12.88***					
	DSE	0.38***	4.14				0.40***	3.40				0.46***	5.25							
	DSS × DSE	0.01	0.15				0.10	0.67				−0.01	−0.07							

*p < 0.05.

**p < 0.01.

***p < 0.001.

In Appreciating others and Being humble, robust t-values were adopted check the significance levels of the parameters because the null hypothesis of homoscedasticity was rejected for either test with a 5% significance level. Otherwise, standard t-values were adopted.

### Factors related to life skills in the action/maintenance stage

Table 3 presents the results of the hierarchical multiple regression analysis models performed to determine DSE and DSS's impact on the association with life skills during the action/maintenance stage. The same factors found in the pre-action stage were added to each model. After adding DSS and DSE to Model 1, the coefficient of determination was significant (*R*^2^ = 0.24, *p* < **0.0**01). Total life skills had a significant positive association only with DSE (β =0.52, *p* < 0.001). In Model 2, when the interaction between DSE and DSS was added, an increment in *R*^2^ was observed (Δ*R*^2^ = 0.06, *p* < 0.05); the interaction term was significantly positively associated with total life skills (β = 0.32, *p* < 0.05). A simple slope analysis was used to examine the identified interaction effect.

**Table 3:** Hierarchical regression results of total life skills and subscales in the action/maintenance stage.

		**Total life skills score**					**Stress management**					**Goal setting**					**Thinking carefully**				
		**β**	***t*-value**	***R* ^2adj^**	** 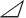 *R* ^2^**	***F*-value**	**β**	***t*-value**	***R* ^2adj^**	** 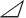 *R* ^2^**	***F*-value**	**β**	***t*-value**	***R* ^2adj^**	** 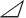 *R* ^2^**	***F*-value**	**β**	***t*-value**	***R* ^2adj^**	** 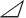 *R* ^2^**	***F*-value**
Step1	DSS	−0.01	−0.05	0.24***	0.26***	10.89***	0.05	0.38	0.14**	0.17**	6.23**	0.09	0.65	0.02	0.05	1.69	0.05	0.34	0.12**	0.15**	5.35**
	DSE	0.52***	4.21				0.39**	2.98				0.17	1.26				0.36**	2.77			
Step2	DSS	−0.19	−1.29	0.28***	0.06*	9.31***	0.07	0.46	0.13*	0.00	4.11*	−0.07	−0.40	0.05	0.04	2.09	0.03	0.20	0.11*	0.00	3.52*
	DSE	0.44***	3.55				0.40**	2.92				0.11	0.75				0.36*	2.60			
	DSS × DSE	0.32*	2.19				−0.04	−0.27				0.28	1.67				0.02	0.14			
		**Appreciating others**					**Communicating**					**Maintaining etiquette and manners**					**Always making one's best effort**				
		β	***t*** **-value**	*R* ^2adj^	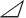 *R* ^2^	***F*** **-value**	β	***t*** **-value**	*R* ^2adj^	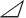 *R* ^2^	***F*** **-value**	β	***t*** **-value**	*R* ^2adj^	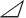 *R* ^2^	***F*** **-value**	β	***t*** **-value**	*R* ^2adj^	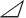 *R* ^2^	***F*** **-value**
Step1	DSS	0.11	0.77	0.01	0.04	1.21	−0.06	−0.44	0.06	0.09	3.09	−0.10	−0.71	−0.02	0.01	0.37	−0.06	−0.44	0.12**	0.15**	5.19**
	DSE	0.12	0.87				0.33	1.98				0.11	0.74				0.40**	3.06			
Step2	DSS	−0.05	−0.29	0.03	0.04	1.72	−0.25	−1.47	0.11*	0.06*	3.61*	−0.18	−1.03	−0.03	0.01	0.45	−0.22	−1.41	0.15**	0.05	4.72**
	DSE	0.06	0.38				0.24	1.58				0.07	0.49				0.33*	2.47			
	DSS × DSE	0.28	1.64				0.34*	2.20				0.14	0.78				0.30	1.84			
		**Taking responsibility for one's own behavior**					**Being humble**					**Maintaining physical health and wellbeing**									
		β	***t*** **-value**	*R* ^2adj^	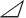 *R* ^2^	***F*** **-value**	β	***t*** **-value**	*R* ^2adj^	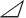 *R* ^2^	***F*** **-value**	β	***t*** **-value**	*R* ^2adj^	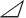 *R* ^2^	***F*** **-value**					
Step1	DSS	0.02	0.17	0.04	0.07	2.21	−0.14	−1.11	0.16**	0.18**	6.87**	−0.03	−0.24	0.11*	0.14*	4.89*					
	DSE	0.25	1.81				0.47**	3.66				0.39**	2.90							
Step2	DSS	−0.05	−0.31	0.03	0.01	1.67	−0.28	−1.84	0.18**	0.03	5.57**	−0.13	−0.78	0.11*	0.02	3.62*					
	DSE	0.22	1.51				0.41**	3.10				0.34*	2.49							
	DSS × DSE	0.14	0.79				0.26	1.62				0.17	1.04							

*p < 0.05.

**p < 0.01.

***p < 0.001.

In communicating, robust t-values were adopted check the significance levels of the parameters because the null hypothesis of homoscedasticity was rejected for either test with a 5% significance level. Otherwise, standard t-values were adopted.

**Figure 2:**
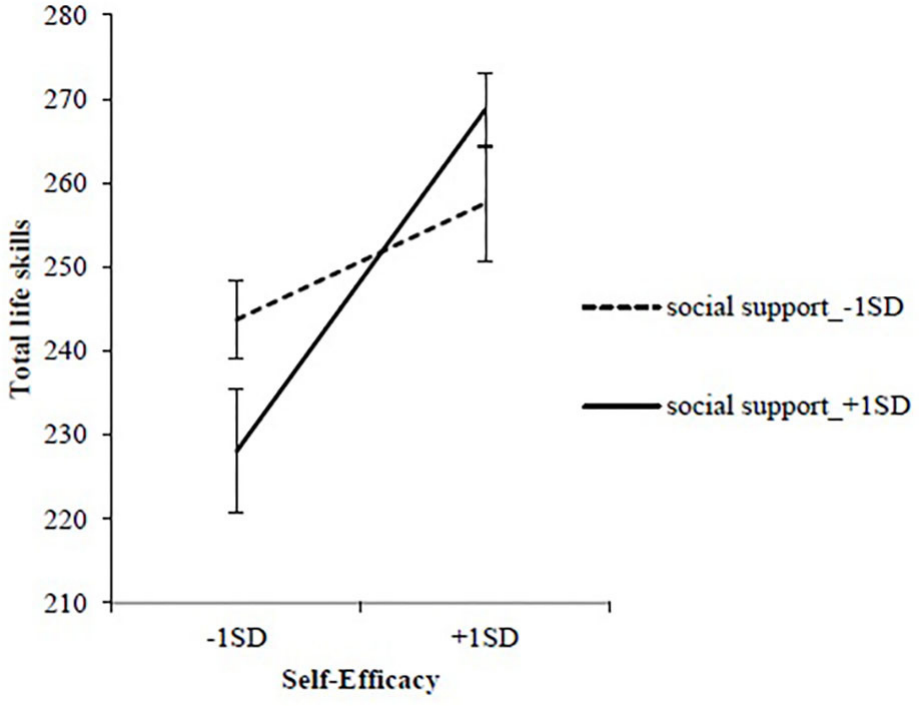
The interaction between self-efficacy and social support related to total life skills in the action/maintenance stage.

**Figure 3:**
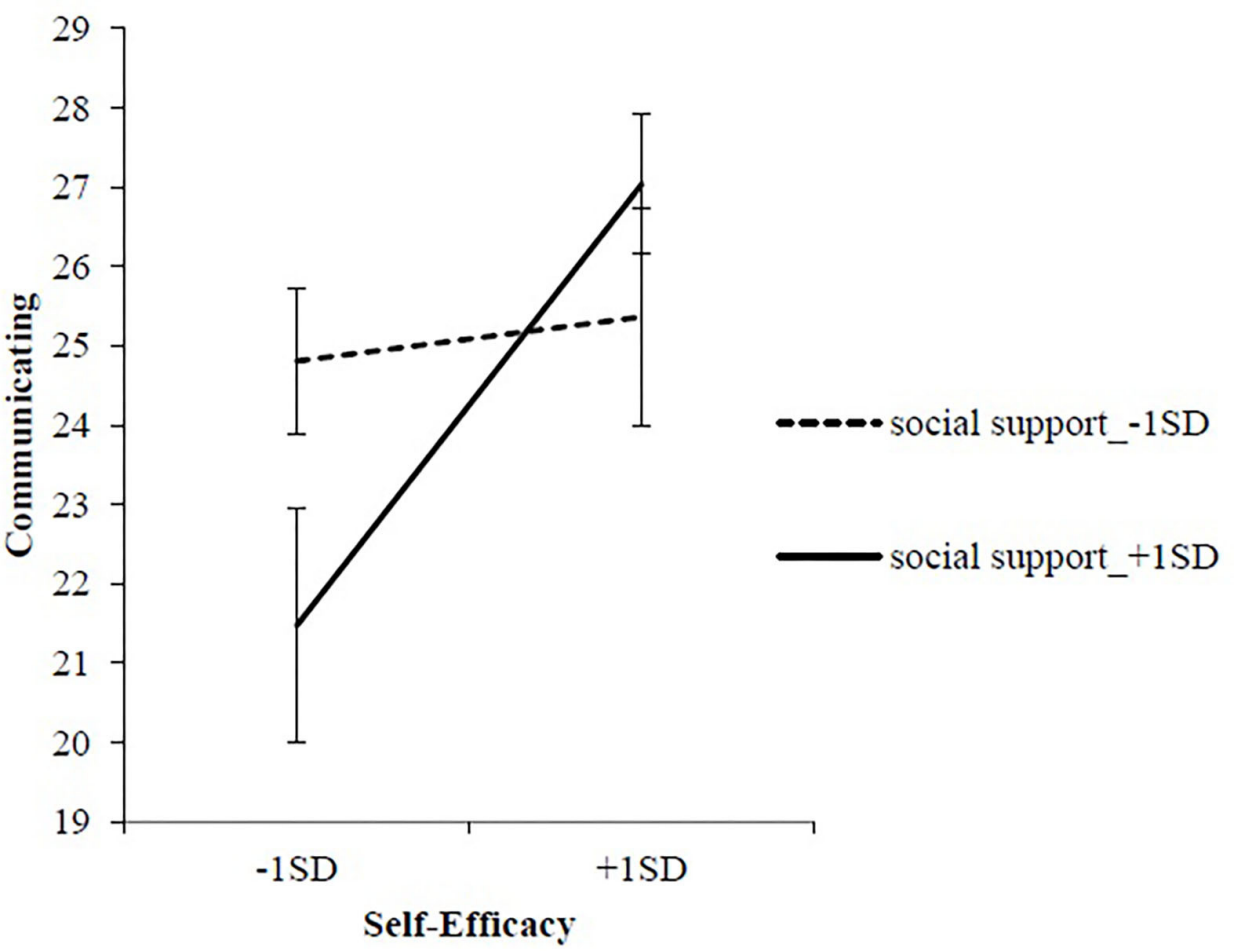
The interaction between self-efficacy and social support related to communicating in the action/maintenance stage.

Figure 2 shows the regression slope for DSS and DSE's influence on total life skills. The slope analysis results indicated that the total life skills score at high DSE was significantly higher than at low DSE in high DSS situations (*p* < 0.001). Upon performing the same steps with life skills subscales as the dependent variables, the increment in *R*^2^ in Model 2 was significant only for the communicating subscale (Δ*R*^2^ = 0.06, *p* < 0.05). The interaction term between DSE and DSS was also significant (β = 0.34, *p* < 0.05). To examine the identified interaction effect, a simple slope analysis was used to compare DSS's impact on DSE in relation to communicating. The results of the slope analysis indicated that the communicating score at high DSE was significantly higher than that at low DSE in situations of high DSS (*p* < 0.01). Conversely, there was no significant difference between the communicating scores in high and low DSE conditions in situations of low DSS (*p* = 0.853; Figure 3). Moreover, the communicating score in high DSS situations was significantly lower than in low DSS situations only when DSE was low (*p* < 0.05; Figure 3).

## Discussion

Based on the stages of change, this study determined the extent to which the association between DSE and DSS impacts life skills acquisition in high school baseball players. Each variable of DSS and DSE independently explained total life skills in the pre-action stage. However, no significant association was found in the interaction terms that combined these explanatory variables.

Contrastingly, there was no significant association between DSS and total life skills among participants in the action/maintenance stage. However, DSE independently explained total life skills, furthermore, the interaction between DSE and DSS were observed. The interaction showed that the total life skills scores for participants with high DSE were significantly higher than the life skills scores for participants with low DSE during instances of high DSS. Thus, while DSS and DSE are independently related to total life skills in the pre-action stage, the combination of DSE and DSS may be related to acquiring total life skills in the action/maintenance stages. Particularly, SE contributes to the operation of the other agentic elements, such as self-regulation through goal setting (29). As DSE explained life skills at both stages of change, it may play a key role in life skills acquisition through dietary behavior, regardless of the stage of change.

In the pre-action stage, no significant association was found between the interaction of DSE and DSS and participants' total life skills. However, the interaction between DSE and DSS did significantly influence the goal-setting subscale. When DSS was high, goal setting at high DSE was significantly higher than at low DSE. Goal setting works to promote dietary change in participants in the nutrition education program (30). In the context of dietary habits, goals are set to manage one's weight and food intake quantity and frequency. Goal setting, required for behavior change or taking initiative, is similar to the self-liberation process of TTM (31). In the TTM, self-liberation is one of the processes of change in the pre-action stages (9). Thus, goal setting may be considered an important skill at this stage.

Additionally, according to the “General guidelines for applying stages and processes of change to adoption of healthful diets,” encouraging an individual to set specific and achievable goals is effective in the preparation stage (32). The importance of receiving feedback from authority figures, along with planning proximal feedback goals is evident (33). Hence, in this stage, it is presumed that DSS is necessary to set food and nutrition-related goals and improve the quality of those goals. Notably, Bandura (34) and Bandura (35) states that efficacy beliefs influence individuals' goalsetting, commitment, and sustaining power of goals in the face of difficulties. This suggests that those with high DSE set challenging goals and develop effective analytical strategies. This study, too, found that with high DSS, the goal-setting score at high DSE was greater than at low DSE. This shows that when DSE is high, the influence of DSS may deepen the analytical thinking component of the participant's goal setting.

Conversely, people who report low SE underestimate their own SE because of the difficulty in acquiring skills owing to their passive attitude toward learning (36). Another possibility is that individuals are actively engaged in learning, but due to their cognitive characteristics, such as high anxiety, they may underestimate their SE because they lack confidence in their ability to accomplish learning tasks (36). Although it is not clear what circumstances apply to the target individual, the results suggest that low SE may inhibit the acquisition of goal setting in either case.

In the action/maintenance stage, no significant association was found between DSS and total life skills. However, when DSS was high, the total life skills score at high DSE was significantly higher than at low DSE. In the action-oriented phase, people increasingly use environmental management and support with the aim of maintaining or terminating behavior (9, 10). In fact, one process encouraging change in the action/maintenance stage of the TTM is “Helping relationships,” including social support for healthy behavior change (9, 10). Notably, there is a demonstrated need for environmental factors in this stage. Self-regulation is also used to sustain behavior: Bandura (4) observes that self-regulatory processes enable people to actively process and transform the environment, rather than simply reacting to them. In other words, at this stage, people do not simply receive SS passively. Instead, they require skills to effectively use SS to improve eating habits and manage their behavior.

The influence of such SS is determined by the recognition of SE, and those with higher SE may be enabled to efficiently manage present internal and external resources (12). Furthermore, active use of feedback positively influences SE (37). Accordingly, individuals with high SE and significant SS can effectively manage this support and increasingly improve their SE. It is presumed that the high score for life skills (including self-regulatory skills) reflects this. Contrastingly, lower SE makes it more difficult to access external measures, including SS (12). Thus, we conclude that when SE is low, SS cannot be employed, and life skills score poorly. This reasoning suggests that the degree of DSE affects the utilization effect of DSS during the action/maintenance stages and that this association between DSE and DSS is possibly related to acquisition of life skills.

In the action/maintenance stage, the interaction between DSE and DSS was not significantly related to goal setting, unlike in the pre-action stage. This means that DSS is not always necessary to set goals at this stage when one is ready for action. Instead of goal setting, the effective interaction of DSE and DSS was observed to influence communication. Furthermore, in situations where a lot of DSS existed, communication was significantly greater at high DSE than at low DSE. Communication skills comprise various factors, including self-control and assertiveness (38). Regarding dietary habits, it is expected that there will be an opportunity to exercise communication and undertake assertive negotiation (such as communicating a meal request to a meal preparer). Although the supporters may encourage such exchanges, it may be necessary for the subjects to work on the external environment themselves. Therefore, repeated practice exchanges with individuals and organizations are necessary to acquire communication skills in dietary situations for those who have dietary DSE to communicate their knowledge and ideas about food to another party.

In contrast, when DSE was low, communication skills were lower when DSS was higher. This reasoning suggests that high DSS may negatively affect the acquisition of communication skills when DSE is low. This observed effect on communication, which requires interpersonal relationship skills among life skills, can be explained by the fact that SS involves relationships with various aspects of the external environment. Since lower SE makes utilization of external resources difficult (12), it is possible that the acquisition of communication skills may be hampered by over-support with extensive interaction in the context of SS. This suggests the need to first consider the degree of DSE when providing DSS—even at the action/maintenance stage.

This study's findings indicate that, among life skills, the acquisition of goal setting (in the pre-action stage) and communicating (in the action/maintenance stage) may be related to adjusting the action of DSS according to the degree of DSE. In conclusion, the acquisition of life skills in dietary situations can be fostered by providing DSS that considers DSE in dietary control according to the stages of change. We recommend that DSS must be appropriate for individuals' degree of DSE when implementing nutrition education.

### Limitations

Since behavioral change was evaluated at the stages of change instead of the behavior itself, it was impossible to know the participants' actual behaviors. Hence, it is necessary to confirm behavioral changes at the behavioral level by focusing on dietary behavior, such as food intake. Baseball is one of the most popular club-sport activities among Japanese high school students (39). Additionally, as of 2021, there are 3,890 teams for boys (40) but only 43 teams for girls (41) in the baseball federation in Japan. Thus, we selected boys as the subjects of this study because there is a greater need for research on male players. Since this study was conducted in a male-dominated sport, high school baseball, only male players were considered, and comparisons with female players could not be made. While the results of this study may also apply to females, we did not examine gender differences in DSE for improving dietary habits in the athlete population. Although a previous study reported that the total DSE scores for improving dietary habits among Japanese high school students, not athletes, did not differ by gender (42), further studies are still needed.

Another potential limitation is that seven of the eight target teams belonged to public schools, and only one team was from a private school. Therefore, it is possible that these results were affected by the socio-economic status of the school. However, this study did not account for the socio-economic status of the individuals and their subsequent results. In addition, the association with communication in the action/maintenance stage may have been recognized because the participants were baseball players. Baseball is a team sport and has the characteristic of being excellently cooperative compared to some other sports (43). Thus, we presumed that the communicative relationships were already clearly present in the team. Therefore, to clarify that the functions of DSS and DSE within communicating are life skills originating in the action/maintenance stage, it is necessary to verify whether similar results can be obtained for sports other than baseball and among adolescents with various backgrounds.

When the validity and reliability of the scale were confirmed, some items did not meet the criteria. However, we decided to retain those items for the sake of generalizability in future studies. Therefore, the validity and reliability of these scale items should be considered while interpreting our results.

### Implications for research and practice

We demonstrated that the degree of DSE and the function of DSS might be related to acquiring life skills that differ depending on the participant's stage of change; for instance, goal setting in the pre-action stage and communicating in the action/maintenance stages. Results indicate that, in the pre-action stage, it is effective to provide DSS associated with goal setting, which motivates individuals with high DSE to commence the action. Therefore, it is necessary to provide DSS, such as by setting an action goal, to inspire behavioral awareness. It is also necessary to promote decision-making that leads to a heightened awareness of the decision to change behavior. Conversely, it may be helpful for those with low SE to first determine the underlying reason for low SE (44), then find a way to strengthen DSE according to the participant's characteristics, and finally, provide DSS for goal setting.

In the action/maintenance stage, it is necessary to provide information about what kind of DSS is available and help people find it, thereby increasing the chances that those with high DSE will receive it. Moreover, participants need to enhance DSS by strengthening their existing social networks or by developing new social network linkages through support groups (3). To this end, one may educate close supporters of participants, such as parents, and create opportunities to engender a new social network in which participants with similar goals can share information. However, when DSE was low, we observed that extensive DSS might negatively affect the acquisition of communication-related life skills. Therefore, we recommend providing DSS that considers the degree of DSE, even in the action/maintenance stage.

Given the cross-sectional design, the relationships between factors related to life skills can only be determined at a single point in time. Accordingly, a longitudinal study is needed to verify causal relationships between DSS efforts and DSE in life skills acquisition.

## Data availability statement

The original contributions presented in the study are included in the article/supplementary material, further inquiries can be directed to the corresponding author.

## Ethics statement

The studies involving human participants were reviewed and approved by the Ritsumeikan University Ethics Review Committee for Medical and Health Research Involving Human Subjects. Written informed consent to participate in this study was provided by the participants and the participants' legal guardian/next of kin.

## Author contributions

YS and KY performed the statistical analysis. YS, KY, and KE contributed to the conception and design of this study. KY, JY, AS, and KE reviewed and edited the manuscript for important intellectual content. All authors have read and approved the submitted version.

## Funding

Ritsumeikan University was funded from the National Federation of Agricultural Cooperative Associations. Data sharing with funders is not applicable in this study.

## Conflict of interest

The authors declare that the research was conducted in the absence of any commercial or financial relationships that could be construed as a potential conflict of interest.

## Publisher's note

All claims expressed in this article are solely those of the authors and do not necessarily represent those of their affiliated organizations, or those of the publisher, the editors and the reviewers. Any product that may be evaluated in this article, or claim that may be made by its manufacturer, is not guaranteed or endorsed by the publisher.

## References

[1] World Health Organization. (1999). Partners in Life Skills Education. Geneva, Switzerland: Department of Mental Health.

[2] World Health Organization. (1994). Life Skills Education for Children and Adolescents in Schools. Geneva, Switzerland: Division of Mental Health.

[3] Contento IR Koch PA eds. (2011). Nutrition Education: Linking Research, Theory, and Practice, 2n ed. Sudbury, MA: Jones & Bartlett Publishers. 18296331

[4] Bandura A. Social Foundations of Thought and Action: A Social Cognitive Theory. Englewood Cliffs, NJ: Prentice Hall (1986).

[5] Bandura A Wood R. Effect of perceived controllability and performance standards on self-regulation of complex decision making. J Pers Soc Psychol. (1989) 56:805–14. 10.1037/0022-3514.56.5.805 2724068

[6] Sheard M Golby J. Effect of a psychological skills training program on swimming performance and positive psychological development. Int J Sport Exerc Psychol. (2006) 4:149–69. 10.1080/1612197X.2006.9671790

[7] Shudo Y Ebi K. Comparison of life skills development between different nutrition education methods for high school baseball players: verification according to the stage of change. J Asia Jpn Res Inst Ritsumeikan Univ. (2021) 3:51.

[8] Gould D Carson S. The relationship between perceived coaching behaviors and developmental benefits of high school sports participation. Hell J Psychol. (2010) 7:298–314.

[9] Prochaska JO Velicer WF. The transtheoretical model of health behavior change. Am J Health Promot. (1997) 12:38–48. 10.4278/0890-1171-12.1.38 10170434

[10] Prochaska JO Redding CA Evers KE. The transtheoretical model and stages of change. In: Glanz K, Rimer B, Viswanath V, editors. Health Behavior: Theory, Research, and Practice, 5th ed. San Francisco, CA: Jossey-Bass Publishers (2015), p. 67–148.

[11] Kim JY Cho SS Kim KW. Psychosocial factors and eating behaviors according to the stages of change in nutrition management among elementary and middle school athletes. Nutr Res Pract. (2021) 15:732–46. 10.4162/nrp.2021.15.6.732 34858551 PMC8601944

[12] Benight CC Swift E Sanger J Smith A Zeppelin D. Coping self-efficacy as a mediator of distress following a natural disaster. J Appl Soc Psychol. (1999) 29:2443–64. 10.1111/j.1559-1816.1999.tb00120.x12092909

[13] Gur K Erol S Kadioglu H Ergun A Boluktas R. The impact on adolescents of a Transtheoretical Model-based programme on fruit and vegetable consumption. Public Health Nutr. (2019) 22:2500–250. 10.1017/S136898001900137X 31171047 PMC10260600

[14] Blow J Sagaribay RIII Cooper TV. A pilot study examining the impact of a brief health education intervention on food choices and exercise in a Latinx college student sample. Appetite. (2022) 173:105979. 10.1016/j.appet.2022.105979 35245642

[15] Selamat R Raib J Aziz NAA Zulkafly N Ismail AN Mohamad WNAW. Fruit and vegetable intake among overweight and obese school children: a cluster randomised control trial. Mal J Nutr. (2021) 27:067–79. 10.31246/mjn-2020-0023

[16] Kim J Kim YM Jang HB Lee HJ Park SI Park KH. Evidence-based nutritional intervention protocol for Korean moderate-severe obese children and adolescents. Clin Nutr Res. (2019) 8:184–95. 10.7762/cnr.2019.8.3.184 31384597 PMC6675960

[17] Jalambadani Z Garmarodi G Yaseri M Tavousi M Jafarian K. The effect of education on reducing fast food consumption in obese Iranian female adolescents - - an application of the transtheoretical model and the theory of planned behavior. Iran Red Crescent Med J. (2017) 19:e13017–22. 10.5812/ircmj.13017

[18] Streda Walker M Dos Santos de Andrades C Schossler Richrot T Closs VE da Silva Gustavo A Da Silva de Oliveira M. Interdisciplinary intervention reduces the consumption of ultra-processed foods in adolescents with overweight or obesity. Saúde Pesqui. (2022) 15:e9700. 10.17765/2176-9206.2022v15n1.e9700

[19] Anderson-Bill ES Winett RA Wojcik JR. Social cognitive determinants of nutrition and physical activity among web-health users enrolling in an online intervention: the influence of social support, self-efficacy, outcome expectations, and self-regulation. J Med Internet Res. (2011) 13:e28. 10.21962196/jmir.1r.5511551 21441100 PMC3221350

[20] Shimamoto K Yonekawa N. The relationships between life skills and competition results in high school golfers. Jpn J Phys Educ Health Sport Sci. (2014) 59:817–27. 10.5432/jjpehss.13049

[21] Shimamoto K Tokairin Y Murakami K Ishii M. Appraisal of required life skills for athletes: a scale development on college student athletes. Jpn J Sport Psychol. (2013) 40:13–30. 10.4146/jjspopsy.2012-1204

[22] Bagozzi RP Yi Y. On the evaluation of structural equation models. J Acad Mark Sci. (1988) 16:74–94. 10.10071007/BF0F02723327

[23] Hair JF Anderson RE Tatham RL Grablowsky BJ. Multivariate Data Analysis. Tulsa, OK: Petroleum Publishing Company (1979).

[24] Fornell C Larcker DF. Evaluating structural equation models with unobservable variables and measurement error. J Mark Res. (1981) 48:39–50. 10.1177/002224378101800104

[25] Haebara T. On the relevance of goodness of model fit to the purpose of research -should we keep the number of indicator variables small? Jpn J Behav. (2002) 29:160–6. 10.2333/jbhmk.29.160

[26] Sato A Sakuma H Kaizaki A Ebi K. Development of stage of change and self-efficacy scales of eating habits for college athletes. Jpn J Sport Nutr. (2017) 10:26–36.

[27] Yamamoto K Akamatsu R Tamaura Y Takemi Y. Functional social support for healthy diets among adult men—analysis of cognitive factors and stage of change for vegetable intake. Ann Inst Nutr Sci. (2011) 17:85–90.

[28] Otaki H Inayama T Nishikawa S. Dietary characteristics and factors related to quality of life in junior, junior high, and high school boys participating in high-level competitive club soccer. Jpn J Nutr Diet. (2012) 70:219–35. 10.5264/eiyogakuzashi.70.219

[29] Bandura A. Toward a psychology of human agency: pathways and reflections. Perspect Psychol Sci. (2018) 13:130–6. 10.1177/1745691617699280 29592657

[30] Cullen KW Baranowski TOM Smith SP. Using goal setting as a strategy for dietary behavior change. J Am Diet Assoc. (2001) 101:562–6. 10.1016/S0002-8223(01)00140-7 11374350

[31] Contento IR Koch PA editors. Nutrition Education: Linking Research, Theory, and Practice, 4th ed. Burlington, MA: Jones & Bartlett Learning Publishers (2020).

[32] Kristal AR Glanz K Curry SJ Patterson RE. How can stages of change be best used in dietary interventions? J Acad Nutr Diet. (1999) 99:679–84. 10.10161016/S0S0002-82-2238223(99)0016500165-0 10361529

[33] Krishna Kumaran SR Yin Y Bailey BP. Plan early, revise more: effects of goal setting and perceived role of the feedback provider on feedback seeking behavior. Proc ACM Hum-Comput Interact. (2021) 5(CSCW1):1–22. 10.1145/3449098

[34] Bandura A. Self-Efficacy: The Exercise of Control. New York, NY: Freeman (1997).

[35] Bandura A. On deconstructing commentaries regarding alternative theories of self-regulation. J Manag Stud. (2015) 41:1025–44. 10.1177/0149206315572826

[36] Sakano Y Maeda M. Serufuefikashii no Rinsho-Shinrigaku [Clinical Psychology of Self-Efficacy]. Kyoto: Kitaojishobo (2001).

[37] Fatima S Ali M Saad MI. The effect of students' conceptions of feedback on academic self-efficacy and self-regulation: evidence from higher education in Pakistan. J Appl Res High Educ. (2021). 10.1108/JARHE-07-2020-0209

[38] Fujimoto M Daibo I. A hierarchical structure theory of communication skills. Jpn J Personal. (2007) 15:347–61. 10.2132/personality.15.347

[39] Sasakawa Sports Foundation editor. The 2019 SSF National Sports-Life Survey of Children and Young People. Tokyo: Sasakawa Sports Foundation (2019).

[40] Japan High School Baseball, Federation. Statistics on the Number of Members. (2021). Available online at: http://www.jhbf.or.jp/data/statistical/index_koushiki.html (accessed September 3, 2021).

[41] Japan High School Girl's baseball Federation. Japan high school girl's baseball Federation list of member schools. (2021). Available online at: http://www.jhgbf.org/jhgbf_kameikou/ (accessed September 3, 2021).

[42] Kibayashi E. Association between self-efficacy and stage of behavior change based on readiness to improve diet in a 3-yearyear follow-up high school study. Jpn J Nutr Diet. (2021) 79:53–63. 10.5264/eiyogakuzashi.79.53

[43] Tokunaga M Yoshida E Shigeeda T Azuma K Inadomi T Saito T. Differences between the sexes, competitive levels and events in the athletes' psychological competitive ability. J Health Sci. (2000) 22:109–20. 10.15017/707

[44] Strecher VJ Seijts GH Kok GJ Latham GP Glasgow R DeVellis B. Goal setting as a strategy for health behavior change. Health Educ Q. (1995) 22:190–200. 10.1177/109019819502200207 7622387

